# Protein kinase C in cellular transformation: a valid target for therapy?

**DOI:** 10.1042/BST20140255

**Published:** 2014-11-17

**Authors:** Anuradha Tarafdar, Alison M. Michie

**Affiliations:** * Paul O’Gorman Leukaemia Research Centre, Institute of Cancer Sciences, University of Glasgow, 21 Shelley Road, Glasgow G12 02D, U.K.

**Keywords:** B-cell receptor (BCR) signalling, chronic lymphocytic leukaemia (CLL), leukaemia, protein kinase C (PKC)

## Abstract

The protein kinase C (PKC) family of serine/threonine protein kinases share structural homology, while exhibiting substantial functional diversity. PKC isoforms are ubiquitously expressed in tissues which makes it difficult to define roles for individual isoforms, with complexity compounded by the finding that PKC isoforms can co-operate with or antagonize other PKC family members. A number of studies suggest the involvement of PKC family members in regulating leukaemic cell survival and proliferation. Chronic lymphocytic leukaemia (CLL), the most common leukaemia in the Western world, exhibits dysregulated expression of PKC isoforms, with recent reports indicating that PKCβ and δ play a critical role in B-cell development, due to their ability to link the B-cell receptor (BCR) with downstream signalling pathways. Given the prognostic significance of the BCR in CLL, inhibition of these BCR/PKC-mediated signalling pathways is of therapeutic relevance. The present review discusses the emerging role of PKC isoforms in the pathophysiology of CLL and assesses approaches that have been undertaken to modulate PKC activity.

## Introduction

The protein kinase C (PKC) family of serine/threonine protein kinases regulate numerous cellular processes including survival, proliferation, differentiation, apoptosis and migration. Members of this family are composed of N-terminal regulatory and C-terminal catalytic domains that are structurally closely related, but can be further divided into three sub-classes based on their structural properties and co-factor requirements: conventional, novel and atypical PKCs (aPKCs). To date, ten distinct PKC isoforms have been identified, encoded by nine genes [1,2]. Conventional PKCs [cPKCs; α, β (alternatively spliced into βI and βII) and γ] contain diacylglycerol (DAG)-binding domains (C1A and C1B) and a calcium (Ca^2+^) binding domain (C2) in the regulatory region and require DAG and Ca^2+^ for activation. Novel PKCs (nPKCs; δ, ε, θ and η) require DAG and phosphatidylserine for activation, but contain a variant of the C2 domain, which is unable to bind Ca^2+^, rendering nPKCs Ca^2+^ independent. aPKCs (ζ and ι (λ in rodents) lack functional binding sites for DAG and Ca^2+^ [3].

Maturation and activation of PKCs require phosphorylation events at AGC kinase conserved sites [2]. In the case of cPKCs, the nascent protein associates with the plasma membrane where it is phosphorylated at the activation loop by phosphoinositide-dependent kinase-1 (PDK1). This event leads to further phosphorylation on the turn and hydrophobic motifs of the PKC, which serve to stabilize the protein, resulting in the release of the mature, primed but inactive, PKC into the cytoplasm [1]. Agonist-induced signals that result in hydrolysis of phosphatidylinositol-4,5-bisphosphate lead to the generation of the second messengers Ca^2+^ and DAG, which enable PKC translocation to the membrane. Engagement of PKC with the membrane leads to a conformational change and full activation of PKC, enabling the activation of PKC-mediated downstream signalling events. The affinity of PKC for the plasma membrane is dependent on the levels of available co-factors, and the maintenance of phosphorylation. Indeed, PKCs are sensitive to dephosphorylation by protein phosphatases such as PHLPP and PP2A, which has an impact on their stability [4]. In addition to translocating to the plasma membrane, PKCs can also translocate to discrete subcellular locations, binding to distinct receptors for activated C-kinase (RACK) proteins, such as the keratin cytoskeleton, tight junctions, Golgi apparatus and the endoplasmic reticulum [5]. Therefore selected translocation of the activated PKCs to specific cellular compartments can assist in the determination of cell fate decisions.

PKC isoforms are ubiquitously expressed in tissues, with studies demonstrating that PKCα, βI & II, δ, ε and ζ are expressed in all major tissues, whereas PKCγ expression is restricted to the central nervous system and spinal cord, PKCη in the skin, lung, spleen and brain, PKCθ in the skeletal muscle, lung, spleen, skin and brain [6–8]. These expression profiles make it difficult to define roles for individual isoforms, with the complexity compounded by the finding that PKC isoforms can co-operate with or antagonize other PKC family members [9,10]. Although genetic targeting of selective PKC isoforms has highlighted a degree of redundancy in the role of PKCs in normal cellular function, this approach has demonstrated the unique role played by individual PKC isoforms in specific cell functions. For example, PKCα has recently been shown to play a critical role in the interleukin (IL)-17A transcription in the Th17 helper T-cell subset, through the regulation of transforming growth factor β receptor 1 (TGFβR1)-mediated phosphorylation of SMAD2-3 [11], whereas the PKCβ-knockout mouse model revealed an immunodeficient phenotype with a pronounced block in B-cell development, reduced numbers of B1 cells and impaired antibody responses [12].

These studies highlight the complexity of PKC isoform regulation at the level of the expression profile within tissues/cells, differential regulation by co-factors and kinases and the distinct expression pattern of RACKs that has an impact on subcellular targeting. Each of these events is highly regulated to generate a signalling response that is unique to the cell/tissue of interest, leading to a finely tuned alteration in cell fate/functional response. In light of this tight regulation, it is perhaps unsurprising that dysregulation of PKC isoforms has been implicated in a plethora of cancers. Alterations in PKC isoenzyme expression have been implicated in transformation events in a number of cell types [13]. Interestingly, the PKCι-knockout mouse is the only model to generate an embryonic lethal phenotype [14]. In the present paper, we review the emerging important role of PKC isoforms in the pathophysiology of chronic lymphocytic leukaemia (CLL).

## Chronic lymphocytic leukaemia

CLL is the most common leukaemia in the Western world, accounting for 1% of new cancer cases in the U.K. and ~35% of all leukaemia types in adults [15]. CLL is characterized by the accumulation of long-lived mature monoclonal B-cells with the distinctive phenotype: CD19^hi^, CD5^+^, CD23^+^, IgM^lo^, FCM7^−^ [15]. Although CLL was originally considered to be a malignancy of defective apoptosis following the discovery that Bcl-2 family of anti-apoptotic proteins were deregulated, it is now established to be a dynamic disorder, with rates of both proliferation and apoptosis of up to 2% of the clone size per day [16]. CLL cell division occurs within ‘proliferation centres’ in the lymph nodes through interaction with the stromal niche antigen and co-stimulation by activated CD4^+^ T lymphocytes expressing CD40 ligand (CD40L), and IL-4 attracted by Ki67^+^ CLL cells [16,17]. Two major prognostic subtypes of CLL are defined by mutational status of variable region within the immunoglobulin heavy chain gene (IgV_H_) of the B-cell receptor (BCR); mutated (M) IgV_H_ genes are associated with favourable outcomes, whereas cases harbouring unmutated (UM) IgV_H_ genes, which can also express ζ-chain (T-cell receptor)-associated protein kinase of 70 kDa (ZAP-70) and CD38, display more aggressive disease and more frequently require therapeutic intervention [18,19]. UM-CLL cells generally retain the ability to transmit signals through their BCR in comparison with M-CLL cells [20], with sustained BCR cross-linking resulting in a phosphoinositide 3-kinase (PI3K)/Akt-mediated up-regulation of Mcl1, which has been shown to increase CLL cell chemoresistance [21–23]. These studies highlight the central role played by BCR-mediated signalling in the pathogenesis of CLL [24]. Therefore a deeper understanding of the signalling pathways that regulate CLL survival and proliferation will highlight key therapeutic targets for inhibition.

## PKC isoforms in CLL

The deregulation of PKC activity/expression in CLL cells was recognized in an early study demonstrating that total PKC activity in CLL was lower than that observed in acute myeloid leukaemia (AML) and acute lymphoblastic leukaemia (ALL) in the particulate and cytosolic fractions; however, a high level of PKCβ expression was associated with CLL in comparison with PKCα and PKCγ [25]. These findings were supported and extended by a study comparing PKC profiles in CLL cells [26]. In comparison with normal mature peripheral-blood-derived B-cells, PKCα and PKCβI expression was down-regulated in CLL cells, whereas PKCβII and PKCε expression was up-regulated. Indeed, PKCβII overexpression in CLL cells was indicated to play a role in CLL pathogenesis, as PKCβII protein levels increase with disease stage and tumour burden [26].

Initial studies assessing the role of PKC in CLL cells utilized reagents such as the macrocyclic lactone bryostatin and the phorbol ester PMA, which globally modulate PKC activities and signalling pathways. In this way, elevated PKC activity was shown to play a role in CLL cell differentiation [27,28], enhanced CLL immunomodulation [29] and increased cell survival and chemoresistance [30,31]. The role of PKC in these processes was inferred by the use of PKC selective inhibitors; however, these studies did not establish the function of individual PKC isoforms. As the complexity of the PKC family has become apparent, subsequent studies have utilized complementary approaches to confirm the role of specific PKC isoforms in CLL cell function.

### PKCα

PKCα expression is down-regulated in CLL cells compared with normal mature B-cells [26]. Interestingly, we had previously shown that inhibiting PKCα activity in B-cell lineage progenitors resulted in the initiation of a CLL-like disease. This was achieved by stably expressing a plasmid-encoding kinase dead PKCα (PKCα-KR) in a haemopoietic stem and progenitor cell-enriched population from wild type mice and culturing these cells in B-cell generation systems *in vitro* and *in vivo*. The resultant cells exhibited hallmark characteristics of human CLL cells at the level of: (i) phenotype, e.g. CD19^hi^ CD5^+^ CD23^+^ IgM^lo^; (ii) cell cycle phase (halted in G_0_/G_1_ *ex vivo*); and (iii) resistance to apoptosis [32]. CLL cells generated *in vivo* accumulated in the lymphoreticular system similar to that noted in CLL patients, demonstrating the applicability of this murine CLL model to the human disease. Our recent work indicates that the PKCα-KR-CLL model exhibits key characteristics of poor prognostic CLL, with cells expressing ZAP-70 and possessing enhanced proliferative capacity both *in vivo* and *in vitro*. Moreover, PKCβII expression is up-regulated during disease progression, suggesting that a functional down-regulation of PKCα signals may generate permissive conditions for the initiation of CLL [33].

### PKCβ

**Figure 1:**
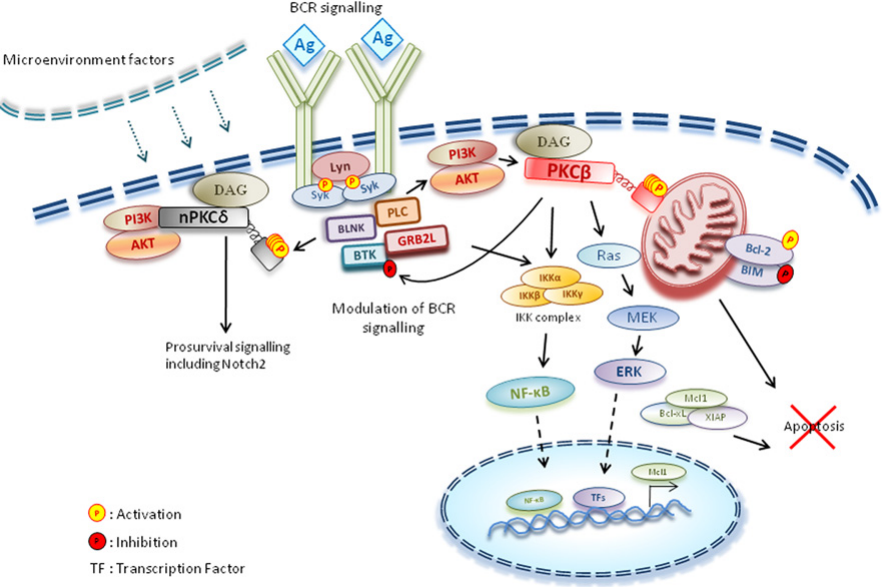
Key events mediated by the BCR BCR signalling regulates B-cell development and function. Coupling of the BCR with PKCβ results in up-regulated MAPK and NF-κB signalling, leading to apoptotic resistance. In addition, PKCβ has been shown to deactivate the non-receptor tyrosine kinase Btk, by phosphorylating Ser^180^ and disrupting plasma membrane targeting. The PI3K-dependent PKCδ is activated by Syk, a tyrosine kinase proximal to the BCR. PKCδ activation is associated with elevated prosurvival signalling such as Notch2 and stabilization of anti-apoptotic factors such as Mcl-1 and XIAP. Ag, antigen; BLNK, B-cell linker; ERK, extracellular-signal-regulated kinase; GRB2L, Grb2 (growth-factor-receptor-bound protein 2)-like; IKK, inhibitor of NF-κB kinase; MEK, MAPK/ERK kinase; PLC, phospholipase C.

The BCR mediates key events during the life cycle of the B lymphocyte to regulate B-cell development and immune cell function. By coupling to the appropriate intracellular signalling molecules, BCR ligation triggers proliferative, survival, differentiation, anergic and/or apoptotic responses. PKCβ has previously been demonstrated to play a critical role in B-cell development, due to its ability to link the BCR with downstream signalling pathways [12]. Indeed, PKCβ has been demonstrated to regulate the nuclear factor κB (NF-κB) signalling pathway downstream of BCR activation [34], in addition to deactivating the non-receptor tyrosine kinase Bruton tyrosine kinase (Btk), by phosphorylating Ser^180^ and disrupting plasma membrane targeting [35] (Figure 1). Given the prognostic significance of the BCR in CLL [24], it is perhaps unsurprising that PKCβ overexpression, specifically PKCβII, is linked to poorer prognostic outcome [26]. PKCβ is also aberrantly up-regulated in other B-lymphocyte malignancies including diffuse large B-cell lymphoma (DLBCL) and mantle cell lymphoma [36,37]. Of note, CLL development in the Tcl-1 transgenic CLL mouse model was abrogated in the absence of PKCβ, supporting a critical role for this PKC isoform in CLL pathogenesis [38].

Assessment of expression levels demonstrated that PKCβII is the dominant PKC isoform expressed in CLL cells, and PKCβII is routinely present in the active form compared with normal B-cells, with membrane-association correlating with its activity. Supporting the role of PKCβ in the negative regulation of Btk, the levels of membrane-associated PKCβII are inversely related to membrane-associated Btk. Moreover, calcium responses after BCR ligation were lower in CLL cells that expressed higher levels of PKCβII [26]. Collectively, these findings demonstrate that PKCβII level/activity plays a central role in modulating signals proximal to the BCR in CLL cells.

PKC- and PI3K-mediated signalling pathways have been established to govern CLL cell survival [21,39]. PKCβ activity can promote phosphorylation/activation of Akt, thus promoting CLL cell survival [40]. In addition, PKCβ regulates Akt by modulating the expression of protein tyrosine phosphatase non-receptor type 22 (PTPN22), a phosphatase that is responsible for positively regulating Akt activity, which is overexpressed in CLL cells [41]. The PKCβ selective inhibitor ruboxistaurin down-regulated PTPN22 and enhanced apoptosis downstream of BCR ligation. Additional mechanisms supporting the pro-survival role of PKCβII are demonstrated in a study showing that PKCβII shuttles to the mitochondrial membrane where it phosphorylates Bcl-2, enabling it to sequester the pro-apoptotic protein BimEL, as well as phosphorylating BimEL and targeting it for proteosomal degradation. These actions skew the CLL cell fate towards apoptotic resistance [42].

Despite expressing high levels of Bcl-2, primary CLL cells require the presence of stromal cells during *in vitro* culture for long-term survival, suggesting that together with their autonomous apoptotic defect, they rely on paracrine signals from the tumour microenvironment [43,44]. PKCβII can also be activated downstream of growth factors such as vascular endothelial growth factor (VEGF) [45], indicating that the activation status of PKCβII and thus its pro-survival function is linked to the lymphoid organ microenvironment of CLL patients. Recently, it was demonstrated that incubation of CLL leukaemic cells with bone marrow stromal cells led to an up-regulation in PKCβII expression and subsequent activation of NF-κB signalling in the stromal cells, which was essential for CLL cell survival [44]. This study emphasizes the complex interplay that occurs between leukaemic cells and their stromal niche and highlights the potential for therapeutic intervention to disrupt both the malignancy and the tumour-associated microenvironment.

### PKCδ

PKCδ is constitutively active in freshly isolated CLL cells. PKCδ activation, as indicated by Thr^505^ phosphorylation, is mediated through a PI3K-dependent pathway [46]. Inhibition of PKCδ resulted in the induction of caspase-dependent apoptosis, and reduction in expression of the anti-apoptotic factors Mcl-1 and X-linked inhibitor of apoptosis (XIAP) [47], suggesting that in addition to PKCβII, PKCδ may play a key role in regulating CLL cell survival. The phosphorylation status of PKCδ has also been linked to spleen tyrosine kinase (Syk), a tyrosine kinase that is proximal to the BCR. Syk activation results in PKCδ-mediated stabilization of Mcl-1, with inhibition of Syk leading to a decrease in PKCδ phosphorylation and an elevation in apoptosis [48] (Figure 1). These studies indicate that PKCδ is associated with CLL cell survival and regulation of Mcl-1 expression, a key antiapoptotic factor associated with chemoresistance [22]. PKCδ is responsible for activation of Notch2 signalling in CLL cells [49]. Increased Notch2 signalling is associated with elevated cell survival in CLL cells [50]. However, the activated Notch2 signal is significantly down-regulated in CLL cells after *in vitro* culture for 24 h, suggesting that the PKCδ/Notch2 signalling pathway is activated in response to microenvironmental stimuli [49].

## Targeting PKCs: current and future directions

As the PKC family has long been considered to play a major role in the pathogenesis of a number of diseases, there has been a focus to develop compounds that selectively modulate the activity of individual isoforms. Therapeutic targeting of individual PKC isoforms represents a significant challenge because PKC isoforms share 70% homology at the ATP binding site, making it extremely difficult to develop isoform-specific drugs [51]. In spite of the initial promise of PKC modulators as a treatment for human diseases, the outcome at clinical trial has been varied and for the most part negative [51]. Most small-molecule inhibitors exhibit undesired off/on target effects making it difficult to translate adequate drug concentrations from animal studies to human clinical trials. Moreover, a lack of pharmacological biomarkers to predict drug efficacy affects the cost and success of clinical trials [51]. Indeed rotterlin, marketed as a PKCδ and Ca^2+^/calmodulin-dependent protein kinase II (CaMKIII) inhibitor, was later shown to also inhibit protein kinases such as checkpoint kinase 2 (CHK2), Polo-like kinase 1 (PLK1) and mitogen-activated protein kinase (MAPK)-activated protein kinase 2 (MAPKAPK2). The cellular effects observed with rotterlin treatment did not correlate with results generated using kinase dead PKCδ, siRNA knockdown of PKCδ or other more selective PKC inhibitors [52].

Early studies showing that bryostatin increased susceptibility of CLL cells to induce apoptosis and/or differentiation [27,28] prompted the clinical assessment of this PKC modulator either alone or in combination with fludarabine or vincristine [53,54]. Patients tolerated bryostatin well, and the *in vitro* findings were supported, with CLL cells undergoing differentiation and a reduction in PKC activity during treatment. Bryostatin was also studied in combination with the anti-CD20 monoclonal antibody rituximab in a Phase II clinical trial (NCT00087425), but the study did not produce data and is now closed. Increased clinical activity of rituximab with conventional chemotherapeutic agents likely made bryostatin-based therapies difficult to justify in CLL.

The first PKC inhibitors used in clinical trial were the staurosporine-derivatives midostaurin (PKC412) and UCN-01 (7-hydroxysporine). Midostaurin was found to be selectively toxic towards the CLL cells compared with peripheral blood lymphocytes *in vitro* [55]. In a Phase II clinical trial, PKC412 was well tolerated by patients, and a reduction in circulating tumour load was noted in more than half of the patients enrolled in the trial and the majority showed a decrease in PKC activity [56]. In addition, PKC412 enhanced cell killing induced by chlorambucil *in vitro* [55]. UCN-01 induced apoptosis in CLL cells both *in vivo* and *in vitro* alone or in combination with fludarabine [57,58]. These studies suggest that targeting PKC may provide a valid therapeutic strategy in CLL and other haematological malignancies.

In CLL, the BCR holds significant prognostic importance, therefore inhibiting the signalling pathways that emanate downstream of BCR ligation are of therapeutic relevance. Indeed, a variety of emerging novel agents are being developed that target BCR signalling components or downstream effectors [59]. Given the evidence supporting a central role for PKCβII in regulating CLL cell survival downstream of the BCR and within tumour microenvironment, this PKC isoform represents a very attractive therapeutic target. Enzastaurin (LY317615), a small-molecule inhibitor targeted towards PI3K/PKCβ pathways, has been demonstrated to induce apoptosis in CLL cells *in vitro* [38,42]; however, there is limited published data available from clinical trials in CLL patients. Enzastaurin showed promise in Phase II clinical trials as monotherapy in DLBCL patients [60]; however, at Phase III in the PRELUDE trial, no clinical significance was observed. It will be interesting to determine whether enzastaurin may be better used as part of a combination therapy, as it has previously been shown to enhance the apoptosis in CLL cells *in vitro* upon combination with fludarabine, vincristine, doxorubicin and bendamustine [42,61]. The use of the PKCβ-selective inhibitor ruboxistaurin (LY333531) in a clinical trial for treatment of leukaemia/lymphoma has not been reported. However, although this inhibitor induced significant apoptosis of CLL cells in response to BCR ligation, survival was unaffected by drug treatment in unstimulated CLL cells [40,41]. These findings indicate that although isoform-selective inhibitors may inhibit certain aspects of disease progression, they will not be curative, suggesting that less-selective PKC inhibitors may hold the key to a promising therapy.

Marked apoptosis has been noted in CLL cells treated with RO32-0432, a pan PKC inhibitor [26]. Sotrastaurin (AEB071), an inhibitor of conventional and novel PKC isotypes, is currently being developed as an immunosuppressive agent for organ transplantation. However, sotrastaurin has been shown to have anti-tumour effects in DLBCL resulting in cell cycle arrest and cell death both *in vitro* and *in vivo* [62] and is currently being used in a Phase I clinical trial for DLBCL and melanoma. Encouragingly, sotrastaurin has recently been shown to display selective cytotoxicity towards CLL cells and can inhibit their proliferation by altering the BCR-dependant survival pathways (MAPK, PI3K and NF-κB). Sotrastaurin not only reduced the levels of survival stimuli within the CLL cells, but also disrupted signals from the microenvironment that contribute to CLL cell survival [63]. Although this latter effect is likely to occur through targeting PKCβII activity, the potential success of pan PKC inhibitors will be due to the inhibition of additional PKC isoforms including PKCδ and PKCε.

As our knowledge of PKC biology deepens, it is evident that specific isoenzyme inhibitors may be insufficient for a drug to have optimal efficacy and minimal off target effects [51]. Indeed, it may be possible to develop drugs that inhibit the interaction of PKC with specific binding partners. This approach requires a detailed understanding of the molecular events that regulate PKC activation. Although the development of separation of function inhibiting peptides that target substrates downstream of a single PKC isoenzyme is at an early stage, peptides have been developed that have biological effect in disease states, holding promise for the future [51]. Finally, early studies suggest that a future approach towards PKC mediated therapies may be to restore PKC activity rather than inhibiting it. One study established that correction of a loss of function point mutation within the regulatory domain of PKCβ in a colon cancer cell line resulted in reduced tumour growth *in vitro* and *in vivo* [64]. Therefore it is important to establish whether modulation of the upstream or downstream targets of the PKC, either inhibition or restoration of activity, would be sufficient to change the course of the malignancy. To achieve this, additional translational research is required: the importance of PKC in cell fate decisions and the limitations of current therapies justify the continued quest for PKC-directed treatments.

## Abbreviations

aPKC: atypical PKC
BCR: B-cell receptor
Btk: Bruton tyrosine kinase
CLL: chronic lymphocytic leukaemia
cPKC: conventional PKC
DAG: diacylglycerol
DLBCL: diffuse large B-cell lymphoma
IL: interleukin
M: mutated
MAPK: mitogen-activated protein kinase
NF-κB: nuclear factor κB
nPKC: novel PKC
PI3K: phosphoinositide 3-kinase
PKC: protein kinase C
PTPN22: protein tyrosine phosphatase non-receptor type 22
RACK: receptor for activated C-kinase
Syk: spleen tyrosine kinase
UM: unmutated
XIAP: X-linked inhibitor of apoptosis
ZAP-70: ζ-chain (T-cell receptor)-associated protein kinase of 70 kDa
